# Entomological survey of the potential vectors of Rift Valley fever virus and absence of detection of the virus genome from the vectors in various niches in the southern half of the Great Rift Valley of Ethiopia

**DOI:** 10.1002/vms3.941

**Published:** 2022-09-14

**Authors:** Megarsa Bedasa Jaleta, Mehari Tefera, Haileleul Negussie, Tesfaye Mulatu, Tsega Berhe, Fasika Belete, Bekele Yalew, Oda Gizaw, Golo Dabasa, Fufa Abunna, Fikru Regassa, Kebede Amenu, Samson Leta

**Affiliations:** ^1^ College of Veterinary Medicine and Agriculture Addis Ababa University Bishoftu Ethiopia; ^2^ Animal Health Institute Entomology Unit Sebeta Ethiopia; ^3^ College of Veterinary Medicine and Agriculture Jimma University Jimma Ethiopia; ^4^ Ministry of Agriculture Livestock and Fishery Addis Ababa Ethiopia

**Keywords:** genome detection, habitats, mosquito, RVF virus

## Abstract

**Background:**

Rift Valley fever virus (RVFV) is the cause of one of the most important mosquito‐borne emerging diseases negatively affecting the health of humans and animals, particularly in Africa. In Ethiopia, the status of RVFV and the existence of potential vectors are unknown.

**Objectives:**

This study aimed to survey the mosquito vectors of RVFV and the detection of the virus in selected sites (Batu, Hawassa, Arba Minch and Borana) in Ethiopia.

**Methods:**

CDC light traps baited with the sugar‐yeast solution were set up at various locations for a total of 29 trap nights. Mosquitoes identification were made morphologically using a stereomicroscope and for RVFV detection by reverse transcriptase‐polymerase chain reaction (RT‐PCR).

**Results:**

Among a total of 132 trap efforts conducted, 60 (45%) captured the mosquitoes. A total of 1576 adult mosquitoes were collected and identified. Including *Aedes* (*n* = 407; 25.8%), *Anopheles* (*n* = 493; 32.3%), *Culex* (*n* = 466; 29.6%) and *Mansonia* (*n* = 210; 13.32%). The genome material of RVFV was not detected by RT‐PCR.

**Conclusions:**

The existence of a potential *Aedes* species may pose a risk for the occurrence of the RVF outbreak in Ethiopia. Based on the current study, we recommend further monitoring for potential mosquito vectors of RVFV, particularly with a view to targeting the seasons during which the mosquitoes can be abundant along with a serological survey of susceptible hosts.

## Graphical abstract

Potential mosquito vectors of Rift Valley Fever virus were collected and identified in Ethiopia, however, the virus genome was not detected from the mosquito pools.

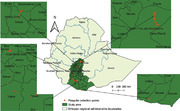

## 1 INTRODUCTION

Mosquito‐borne viral diseases are becoming a major public and animal health challenge (Cooper et al., 2015; Wilder‐Smith et al., 2017). Rift Valley fever (RVF) is a mosquito‐borne viral disease with veterinary and public health implications (Pepin et al., 2010; Nanyingi et al., 2015). The causative agent of the disease is the RVF virus (RVFV) of the genus *Phlebovirus*, family *Phenuiviridae*, order *Bunyavirales* (Abudurexiti et al., 2019). Historical perspective showed that the virus was first identified in 1930, when it caused an outbreak of sudden mass deaths and abortions in sheep around Lake Naivasha in the Greater Rift Valley of Kenya (Daubney et al., 1931). Subsequently, outbreaks of RVF have been reported in some other African countries (Davies, 2010) such as Egypt in 1977, Madagascar in 1979 and Mauritania in 1989 (Pepin et al., 2010) and more recently in 2000 in Middle East countries such as Saudi Arabia and Yemen (Miller et al., 2002). In general, RVF is widespread in Africa where 36 African countries have reported cases of the disease (Leta et al., 2018).

RVFV has a wide range of natural hosts, of which the virus infection was reported from domestic ruminants and camels (Wright et al., 2019; Tchouassi et al., 2016), many wildlife (Rostal et al., 2017), and humans (LaBeaud et al., 2008). There are differences in the susceptibility to the virus in domestic animals with sheep being highly susceptible (Mansfield et al., 2015). RVFV is adapted to a variety of mosquitoes (Arum et al., 2015), and mammals usually acquire the viral infection through mosquito (Diptera: *Culicidae*) bites. In regions where RVF epidemics occurred, the virus has been isolated from at least 53 mosquito species which are grouped into eight genera (Linthicum et al., 2016). The mosquito vectors that transmit RVFV are classified into two major groups, namely primary and secondary vectors (Arum et al., 2015). Some mosquitoes of the genus *Aedes* (also called floodwater mosquito) are considered to be the primary maintenance host and source of RVFV that initiates disease outbreaks (Himeidan et al., 2014; Arum et al., 2015; Sang et al., 2017). The genera *Culex*, *Eretmopodites* and *Mansonia* constitute the secondary vectors with an affinity for flooded grounds to lay their eggs. Mosquitoes in this group, due to their ubiquitous bite patterns, contribute to the amplification of the virus, which subsequently leads to disease outbreaks (USG agencies Working Group, 2015; Arum et al., 2015). RVFV is also mechanically transmitted by other arthropods such as *Culicoides*, sand flies and *Stomoxys calcitrans* (Hoch et al., 1985; Turell et al., 2010).

The epidemiology of RVFV is highly dynamic and intricate under which effective transmission mainly depends on various factors such as the availability of competent vectors, susceptible hosts, and suitable ecological and environmental conditions that support mosquito survival and reproduction (Chevalier, 2013; Iaconoa et al., 2018; Arum et al., 2015). Once the virus is brought into favourable ecologies, it becomes endemic with periodic outbreaks and can spread even further into non‐endemic environments of permissive areas (Baba et al., 2016).

RVF has notable socioeconomic and public health impacts associated with high mortality and morbidity in livestock and human with subsequent negative economic impact through livestock trade ban (Muga et al., 2015). Countries in the Horn of Africa have been affected by RVF. Kenya in particular has experienced multiple outbreaks, with about 23 outbreaks reported between 1912 to 2007 that resulted in several human fatalities and the loss of large numbers of domestic ruminants (Baba et al., 2016; Mosomtai et al., 2018, 2016). RVF causes economic losses due to the ban on livestock trade in the countries where income mainly depends on livestock markets. For example, during the 2006–2007 RVF outbreak in East Africa, an estimated loss of more than US$60 million was recorded associated with livestock trade disruptions (Himeidanet al., 2014). In the Somali region of Ethiopia, GDP was reduced by about 25% due to the trade embargo imposed on the country during the 1998–2000 outbreak in East African countries (Nin Pratt et al., 2005) and similarly about 5% GDP reduction during the 2006–2007 RVF outbreak in Somalia country (Wright et al., 2019).

During outbreaks, the disease affects a large number of animals and is widespread in permissive areas. For example, the outbreak in the year 2007 affected livestock in 11 provinces in Tanzania and Kenya, resulting in the deaths of 16,973 cattle, 20,193 goats and 12,124 sheep with abortion reported in 15,726 cattle, 19,199 goats and 11,085 sheep (Himeidan et al., 2014). Similarly, in Tanzania alone, about 21 out of 28 districts were affected in the 2006–2007 RVF outbreaks, while in Kenya the outbreaks affected 38 out of 69 districts (Baba et al., 2016). Although Ethiopia shares long borders with disease‐endemic countries such as Kenya, Somalia and Sudan (Tran et al., 2016), there have been no reports of cases of RVF from Ethiopia except for some recent serological evidence (Ibrahim et al., 2021; Endale et al., 2021; Asebe et al., 2020).

According to a forecast model developed by Weledekidane (2018) using localised seasonal precipitation data, the Horn of Africa precipitation anomaly pattern coincided with the 1997/1998 and 2006/2007 RVF outbreaks in the region. The model also showed a similar RVF‐favourable climatic situation over the southern and south‐eastern lowland parts of Ethiopia. Similarly, Tran et al. (2016) showed that the northern and southwestern parts of Ethiopia are at higher risk of RVF due to geographic proximity and generally evident cross‐border livestock movement. The East African El Niño warm events of 2015–2016 caused climate and environmental anomalies that led to the outbreak of RVF in many countries in the region (Anyamba et al., 2019). However, there was no report of an RVF outbreak in Ethiopia during the events.

Considering the huge health and economic impacts of RVF as well as the high potential of the expansion of the disease to non‐endemic areas, regular surveillance and prediction are important. Specifically, the implementation of regular surveillance can greatly contribute towards designing an effective mechanism of the control and prevention of the disease (Wilson et al., 2014; Chevalier, 2013). Surveillance of the diseases, particularly in the part of the country shared borders with the neighbour endemic countries where information about the disease is insufficient or absent is particularly useful for control (Weledekidane, 2018; Tran et al., 2016). Similar to other mosquito‐borne diseases, identification of potential mosquito species transmitting RVF is an important step that can help to acquire biological information such as oviposition sites, biting and resting habits that differ among mosquito species (Taira et al., 2012).

Some entomological surveys were conducted after the outbreak of other mosquito‐borne viral diseases in Ethiopia. For example, during the yellow fever outbreak in the South Omo Zone from November 2012 to October 2013, a vector survey was conducted and *Aedes aegypti* was the most abundant among the mosquitoes collected (Lilay et al., 2017). Similarly, after dengue outbreaks in Dire Dawa, eastern Ethiopia (Getachew et al., 2015) and Metema and Humera, north Ethiopia (Ferede et al., 2018), mosquito larvae were collected and morphologically identified. However, an entomological study regarding the presence and distribution of potential vectors of RVFV has not yet been investigated. Therefore, this study aimed to investigate the potential mosquito vector of RVFV in different geographic niches and also to perform the molecular detection of the virus in the mosquitoes.

## 2 MATERIALS AND METHODS

This study focused on an entomological survey of mosquito vectors potentially transmitting RVFV at seven different specific sites in Ethiopia. In particular, the sites included in the present study can be grouped into two: (1) those sites supposed to favour the introduction (e.g., due to geographic proximity to the endemic areas) and (2) those locations considered to be significant maintenance for the vectors of RVFV (e.g., near bodies of water). The mosquitoes collected from different sites using CDC traps were also tested for the presence of the RVFV using RT‐PCR.

### 2.1 Mosquito collection areas

**FIGURE 1:**
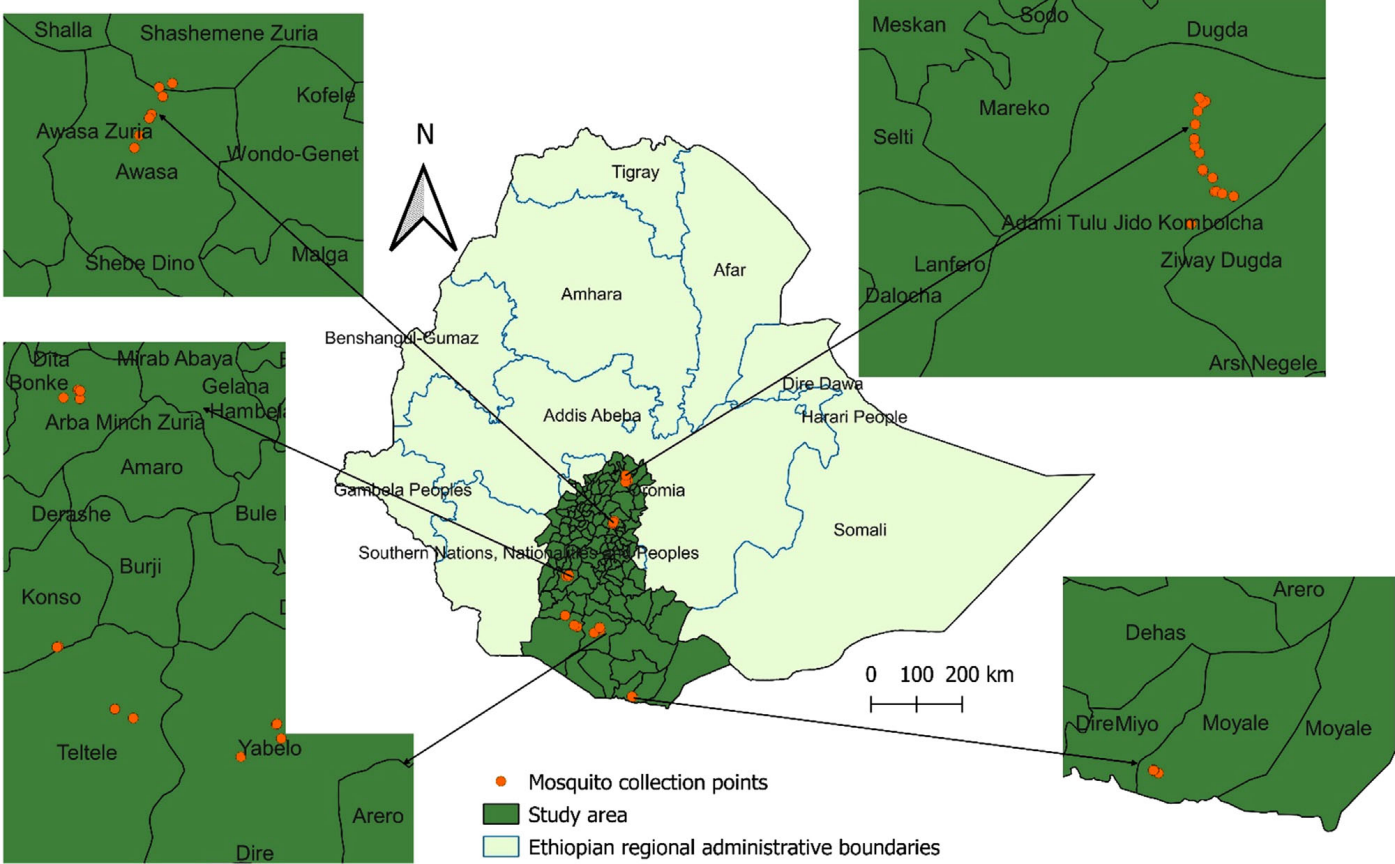
Study area and mosquito collection sites

This study was designed to investigate the mosquito vectors of RVFV in the southern half of the Great Rift Valley area of Ethiopia. Three specific sites (Moyale, Elweya, Yabello) in Borana, southern Ethiopia were included in the present study taking into account the fact that the area shares a border with the RVF‐endemic country of Kenya and there is apparent unrestricted high livestock mobility across the shared borders. The other sites considered were areas around Batu (in Adami Tullu Jido Kombolcha district), the cities of Hawassa and Arba Minch and Konso. This was done to include large permanent bodies of water such as Lake Batu, Lake Hawassa, Lake Abaya around Arba Minch and the Segen Valley river basins in the Konso Special District (Figure 1). The locations given are believed to support oviposition and development of mosquito larvae year‐round. Figure 1 shows the study area and then zooms in and marks the mosquito collection sites. Unfortunately, because the grant for the study was financed too late to conduct work during the wetter seasons, the entomological survey was conducted during the dry season from December 2018 to April 2019 and encompassed the areas around the water bodies. The specific geographic points where the traps were placed are presented as a supplemental file (Supplemental data).

### 2.2 Mosquito collection and identification

Mosquito collections were conducted in three rounds; the first round from 2 to 15 December 2018 around Batu and Hawassa lakes, the second from 14 to 28 February 2019 around Arba Minch and the third phase from 10 to 24 April 2019 in the Borana zone and Segen valley. Mosquitoes were collected using CDC light traps baited with a sugar‐yeast solution. The traps were placed near potential mosquito oviposition and feeding sites, including indoor and outdoor areas, particularly near the water bodies, near the animal enclosure, and in the field where dense human and livestock populations existed.

Mosquitoes have diverse feeding behaviours, with some members of the genus *Aedes* being diurnal (biting mainly in the morning or evening) while others are nocturnal, while most members of the genera *Anopheles*, *Culex* and *Mansonia* are nocturnal feeders (Rozendaal, 1997). To accommodate the different feeding times of the mosquitoes, some of the traps were placed at 18:00 and collected between 6:00 and 7:00 h on the next day while others were set at around 16:00 and collected at 9:00 (the next day). The collection cups were deep frozen (–20°C) for 15 min to tranquillise the mosquitoes for preservation. In the laboratory, sorting and identification of the genus and species levels was performed using dichotomous keys from the Walter Reed BioSystemics Unit (WRBU) (Potter, 2016) and Edwards (Edwards, 1941).

The morphology of the mosquitoes was carefully observed by an entomologist using a stereomicroscope. Some of the mosquitoes identified were used for molecular detection of RVFV. At least one pooled sample consisting of 22–25 female mosquitoes was collected from each collection site and preserved within an hour of collection. For the detection of RVFV, the genera *Aedes*, *Anopheles, Culex* and *Mansonia* were pooled with particular attention given to the genus *Aedes* (due to the prominent role of mosquito species within this genus in initiating and maintaining RVFV).

The pools have different combinations with some of them containing specific mosquito species, while other pools consist of different species of the same genus and/or other genera. The selected mosquitoes identified were placed in a 2 ml Eppendorf tube labelled with information regarding the collection date and trap number. Thereafter, the pool was transferred to a liquid nitrogen container and transported to the National Veterinary Institute (NVI), Bishoftu, and then the samples were stored at –80°C until processing for molecular detection.

### 2.3 Viral genome extraction and amplification

At NVI's laboratory of, the preserved mosquito pools were processed to identify the presence of the RVFV genome in mosquitoes. For the purpose of extraction, each pool was grounded using a sterile mortar and pestle by adding 2–3 ml of phosphate buffered saline (PBS) containing antibiotics (penicillin, streptomycin and gentamycin). After centrifugation at 12,000 rpm for 10 min, the supernatant was aliquoted and transferred to a 1.5 ml cryotube and stored at –20°C until further testing. Then RNA extraction was performed using RNAeasy Mini‐Kit for Nucleic Acid Extraction (QIAGEN, AMBION, Inc., Austin, Texas, USA) according to the manufacturer's instructions. As this was the first RVFV detection attempt in mosquitoes in Ethiopia, isolation control was required to detect false negative results. Unfortunately we could not get an RNA spike‐in kit for this purpose.

The virus detection test was performed in mosquito pools using RT‐PCR. Amplification was performed using specific primers (forward primer *S432*: 5’‐ATGATGACATTAGAAGGGA‐3’ and reverse primer *NS3m*: 5’‐GATGCTGGGAAGTGATGAG‐3’) targeting the S segment region of the viral genome to amplify 298 bp according to the procedure described elsewhere (Garcia et al., 2001). RT‐PCR was performed using a one‐step RT‐PCR kit (Invitrogen, USA) and the master mixes were prepared according to the manufacturer's instructions.

The cycling program consisted of a reverse transcription step at 50°C for 30 min. An initiation denaturation step at 95°C for 15 min was performed to inactivate excess reverse transcriptase enzyme. This was followed by 30 cycles of denaturation at 94°C for 30 s, annealing temperatures at 40°C for 30 s each and extension at 72°C for 30 s. The reaction mixture in each PCR tube was then subjected to a single cycle of the final extension step at 72°C for 5 min. All PCR amplifications were performed with a volume of 50 μl per tube. The positive control for RVFV was kindly provided by the African Union Panafrican Veterinary Vaccine Center while nuclease‐free water was used as the negative control. The RT‐PCR products were visualised by an electrophoresis gel documentation system and scored based on the size of the PCR products.

## 3 RESULTS

### 3.1 Mosquito survey

Out of a total of 132 traps placed, it was possible to catch mosquitoes in 60 traps at different locations (Table 1). A total of 29 nights of trapping were conducted and the number of trapping nights per site varied. The number of traps placed ranged from 6 to 37 per site, with the lowest number being placed around the Segen River and the highest around Lake Batu. During the study period, the average day and night temperatures in the study areas were also assessed as follows: Batu (27°C and 15.5°C) and Hawassa (27°C and 12°C) in December 2018; Arba Minch (30°C and 19°C) in March 2019, Moyale (25°C and 18°C) and Konso River Valley (28°C and 18°C) in April 2019. A total of 1576 adult mosquitoes were collected and identified. The mosquitoes identified belonged to the genus: *Aedes* (*n* = 407; 25.8%), *Culex* (*n* = 466; 29.6%), *Mansonia* (*n* = 210; 13.3%) and *Anopheles* (*n* = 493; 31 .3%). There were many mosquito body parts in trap cups that could not be identified because they were severely damaged and morphologically distorted. At the species level, the major species collected and identified were *Mansonia uniformis*, *Ae. ochraceus*, *Ae. geniculatus*, *Cx. Antennatus* and *Cx. univitattus* as shown in Table 2. The percentage of mosquitoes identified per site was 37.5% (*n* = 584) in Borana, 11.9% (*n* = 187) in Lake Batu, 22.9% (*n* = 361) in Lake Hawassa, 21.6% (*n* = 341) in Lake Abaya and 6.5% (*n* = 103) in the Segen River.

**TABLE 1:** Number of trap effort, trap nights and captures across sites in an entomological survey of RVFV mosquito vectors in Ethiopia

					Habitats		
Sites	Number of nights trapping attempted	Total number of traps placed per site	Total number of trap captured mosquito	Per cent of traps captured mosquitoes	Near water	Indoor	Outdoor
Batu (Lake Batu)	7	37	20	54%	19	7	11
Arba Minch (Lake Abay)	5	26	9	35%	16	3	7
Borana							
Moyale	3	13	4	31%	6	2	5
Elweya	2	8	5	63%	‐	3	5
Yabello	5	21	5	24%	12	1	8
Hawassa (Lake Hawassa)	5	21	15	71%	8	2	11
SegenValley (Segen river)	2	6	2	33%	6	‐	‐
Total	29	132	60		67	18	47

**TABLE 2:** Mosquito species collected across the study sites

Mosquito species	Moyale	Yabello	El‐weye	Batu	Arba Minch	Hawassa	Segen Valley	Total number and %
*Ae. geniculatus*	5	6	13	‐	14	18	4	60 (3.8%)
*Ae. mcintoshi*	5	‐	‐	‐	‐	‐	‐	5 (0.3%)
*Ae. ochraceus*	92	6	4	‐	9	‐	15	126 (8.0%)
*Ae. vexans*	13	4	9	2	‐	4	‐	32 (2.0%)
*Other Aedes species*	58	15	24	4	53	23	7	184 (11.7%)
*Anophilus Arabiensis*	19	10	‐	9	16	‐	‐	54 (3.4%)
*An. gambiae*	8	2	12	7	‐	6	29	64 (4.1%)
Other *Anopheles species*	16	28	15	89	77	116	34	375 (23.8%)
*Cx. antennatus*	14	‐	14	‐	‐	92	‐	120 (7.6%)
*Cx. pipiens*	9	4	11	‐	‐	34	6	64 (4.1%)
*Cx. quinquefasciatus*	115	13	6	‐	‐	‐	8	142 (9.0%)
*Cx. theileri*	17	‐	‐	18	‐	‐	‐	35 (2.2%)
*Cx. univitattus*	17	‐	‐	‐	82	6	‐	105 (6.7%)
*Mansonia uniformis*	‐	‐	‐	58	90	62	‐	210 (13.3%)
Total	388	88	108	187	341	361	103	1576
%	24.6%	5.6%	6.9%	11.9%	21.6%	22.9%	6.5%	100.0%

The number of mosquitoes collected around water bodies was 1099 (69.73%), higher than other places as shown in Table 3. Of the mosquitoes identified, a total of 797 belonged to four genera, *Aedes* (291), *Anopheles* (99), *Culex* (244), *Mansonia* (163), were combined into 32 pools.

**TABLE 3:** Mosquito species collected from different habitats

Mosquito species	Indoor	Near water	Outdoor	Number and per cent	References^†^
*Ae. mcintoshi*	‐	5	‐	5 (0.3%)	Pepin et al. (2010)
*Ae. ochraceus*	‐	116	10	126 (8.0%)	Pepin et al. (2010)
*Ae. vexans*	4	17	10	31 (2.0%)	Tantely et al. (2015)
*Ae. bromeliae*	‐	8	‐	8 (0.5%)	
*Ae. cumminsii*	‐	4	‐	4 (3.0%)	Tantely et al. (2015)
*Ae. geniculatus*	‐	48	12	60 (3.9%)	
*Ae. furcifer*	6	11	2	19 (1.2%)	
Other *Aedes species*	4	105	34	143(9.7%)	
*An. arabiensis*	8	37	9	54 (4.0%)	Tantely et al. (2015)
*An. gambiae*	5	56	3	64 (2.9%)	Tandina et al. (2018)
Other *Anopheles species*	47	299	40	386 (24.5%)	
*Cx. antennatus*	70	26	24	120 (7.6%)	Linthicum et al. (1985), Pepin et al. (2010), Tandina et al. (2018)
*Cx. univitattus*	3	97	5	105 (7.0%)	Tandina et al. (2018)
*Cx.quinquefasciatus*	13	124	5	142 (9.0%)	Tandina et al. (2018)
*Cx. pipiens*	34	19	11	64 (4.1%)	Pepin et al. (2010)
*Cx. theileri*	15	14	6	35 (2.2%)	Pepin et al. (2010)
*Mansonia uniformis*	56	113	41	210 (13.3%)	Tantely et al. (2015)
Total	265	1099	212	1576	
%	16.8%	69.7%	13.5%	100.0%	

^†^ References from which information obtained regarding vector potentially transmitting RVFV.

### 3.2 Viral detection

Of 32 mosquito pools with 20–25 female mosquitoes per pool processed for viral nucleic acid detection by RT‐PCR, none of the pools were positive for the RVFV genome.

## 4 DISCUSSION

The current entomological survey was conducted to assess the occurrence of RVFV mosquito vectors of the in different niches in the southern half of the Great Rift Valley region in Ethiopia. The study was considered an important step in assessing and describing the risk level of RVF in Ethiopia. The survey revealed the existence of several mosquito species with a large number of competent and potential vectors of RVFV in the catches.

*Aedes* mosquitoes were incriminated as principal vectors for RVFV and believed to play a significant role in maintaining the endemicity of the disease in the environment through transovarian transmission (Alhaj et al., 2017). Two of the known competent RVFV vectors in East Africa (*Ae. mcintoshi* and *Ae. ochraceus*) were caught and identified in the present study. In Kenya, studies showed a predominant occurrence of these two species and have been implicated as a reason for the initiation and spread of RVFV during disease outbreaks (Arum et al., 2015; Ochieng et al., 2016; Sang et al., 2017). Mosquitoes of the genus *Culex* have also been considered as potential vectors as a due to their bioecology in terms of abundance, biting activity, feeding habits and longevity (Brustolin et al., 2017). RVFV has been detected in many species of these mosquitoes in Madagascar (Tantely et al., 2015). In the present study, five *Culex spp*. (Table 2) have been identified and if the disease breaks out at the sites, there is a possibility that the disease will spread widely. Similarly, two *Anopheles* species (*Anopheles arabiansis* and *Anopheles gambiae*) were identified in the current study. These *Anopheles* species have been found to be infected with RVFV in Sudan (Seufi and Galal, 2010) and Kenya (Sang et al., 2017).

Mosquitoes typically search areas near bodies water to lay eggs (Che et al., 2013) and therefore, the occurrence and abundance of RVFV mosquito vectors are influenced by the type of biotope (temporary ponds, river or lakes) (Biteye et al., 2018). In general, Borana is an arid and semi‐arid environment with minimal rainfall. A pond is the common body of water in the area, which is surface water harvested from rain in valleys. In Borana, a community pond is made by blocking floods in valleys when it rain or constructing a dam along a dry river during the dry season to collect the upcoming rainwater during the rainy season (Godana and Derib, 2021). During the current study period, these ponds have been reduced to less than their full size (full dam). Many *Aedes* and *Culex* mosquito species, mainly *Ae*. ochraceus and *Cx. quinquefasciatus* have been collected around the ponds, particularly in Moyale District. A study showed that *Ae. ochraceus* and *Ae. mcintoshi* played prominent role in the RVF epidemic in Kenya in 2006/2007 (Sang et al., 2017). Others such as *Cx. pipiens*, *Cx. antennatus* and *Ae. mcintoshi*, which are competent vector of RVFV (Turell et al., 2008), have also been identified in the catches from Borana. The presence of potential vectors in this area, coupled with the nature of transboundary livestock movement (Lasage et al., 2010) and proximity to an endemic area, particularly Marsabit County in Kenya (Hassan et al., 2020), indicated the possibility of RVF outbreak could happen in the border area with Kenya.

The present study did not detect RVFV in the tested mosquitoes. However, there are several limitations that need to be considered, so the results should be interpreted cautiously. Unfortunately, research funding was limited and this forced us to conduct most of the study near bodies of water during the dry season rather than during the more appropriate wet season. This resulted in only a small sample size of mosquitoes being collected and potential vectors may not have been captured and identified. Also, in the current study, we used a yeast‐sugar solution as a CO_2_ source to attract mosquitoes. This was because we could not sustain dry ice in field situations. Although yeast‐generated CO_2_ is a convenient carbon dioxide source for mosquito trapping (Smallegange et al., 2010; Jerry et al., 2017), it is not as standardised as commercial CO_2_; For example, the flow rate of CO_2_ and the effects of other gaseous products are unknown and may have influenced mosquito collection.

The virus could circulate in the sub‐detection level and spread if favourable conditions are met (Pepin et al., 2010). While no cases of RVF in humans or animals have been reported in Ethiopia, there have been reports of serological evidence. Blood samples obtained from livestock in the Adadle district of the Somali region (Ibrahim et al., 2021), the South Omo area (Endale et al., 2021) and the Lare district of the Gambella region bordering South Sudan (Asebe et al., 2020) showed seropositivity to RVFV. Studies conducted in many other countries show that RVFV can circulate in apparently healthy animals without recognised clinical sign of disease, and that once introduced, RVF virus persists for long time, particularly in areas where mosquito multiplication is supported (Lichoti et al., 2014; Ibrahim et al., 2021; Lumley et al., 2018). Given the presence of potential vectors and circulating antibodies in Ethiopia, the possibility of an RVF outbreak cannot be excluded.

RVFV is transmitted to animals by the bites of infected mosquitoes, particularly those of the genera *Aedes*, *Culex* and *Mansonia* (Che et al., 2013). In contrast to the current absence of viral genome in mosquitoes, RVFV was detected from many similar mosquito species during the outbreak in Madagascar (Ratovonjato et al., 2011), Sudan and Egypt (Seufi and Galal, 2010), Kenya (Linthicum et al., 1985) and in experimental infection in Europe (Brustolin et al., 2017). However, during the inter‐epidemic period, the probability of detecting the RVFV genome in mosquitoes is extremely low (Mhina et al., 2015; Pachka et al., 2016; Alhaj et al., 2017).

In conclusion, although no clinical cases of RVF have yet been observed and detection of the virus has not been reported in Ethiopia, (1) the presence of diversified competent RVFV mosquito vectors, (2) the country's geographical proximity to RVF‐endemic countries and (3) the nature of livestock movements across the international border may lead to the conclusion that Ethiopia is at risk of RVF outbreak during epidemic periods in the Horn of Africa. Our findings provided up‐to‐date information that will help to set up efficient entomological and RVF disease surveillance and intervention strategies during high mosquito activity, particularly along border areas with endemic countries.

## AUTHOR CONTRIBUTIONS

Megarsa Bedasa Jaleta: Conceptualisation; data curation; investigation; methodology; software; validation; visualisation; writing – original draft; writing – review & editing. Mehari Tefera: Investigation; writing – original draft. Haileleul Negussie: Conceptualisation; funding acquisition; methodology; supervision; writing – original draft; writing – review & editing. Tesfaye Mulatu: Data curation; investigation; project administration; resources. Oda Gizaw: Conceptualisation; data curation; investigation; methodology; writing – original draft. Golo Dabasa: Conceptualisation; data curation; investigation; methodology; writing – original draft. Fufa Abunna: Conceptualisation; supervision; writing – original draft. Fikru Regassa: Conceptualisation; methodology; project administration; resources. Kebede Amenu: Conceptualisation; funding acquisition; investigation; methodology; supervision; writing – original draft; writing – review & editing. Samson Leta: Conceptualisation; data curation; funding acquisition; investigation; methodology; project administration; resources; supervision; validation; writing – original draft; writing – review & editing.

## CONFLICT OF INTEREST

The authors have declared that they have no conflict of interests.

### ETHICAL STATEMENT

Ethical clearance was obtained from the Animal Research Ethics Review Committee of the Addis Ababa University College of Veterinary Medicine and Agriculture. No. VM/ERC/02/06/10/2018. Informed verbal consent was also obtained from all households participating in the study.

### PEER REVIEW

The peer review history for this article is available at https://publons.com/publon/10.1002/vms3.941.

## Supporting information

Supporting Information

## Data Availability

The data supporting the findings of this study are available within the supplementary material along with this article (Supplemental data).
